# Supplementary figures and images for: RIDDEN: Data-driven inference of receptor activity from transcriptomic data
Source: PLoS Comput Biol. 2025 Jun 16;21(6):e1013188. doi: 10.1371/journal.pcbi.1013188 (PMC12193052; doi:10.1371/journal.pcbi.1013188)

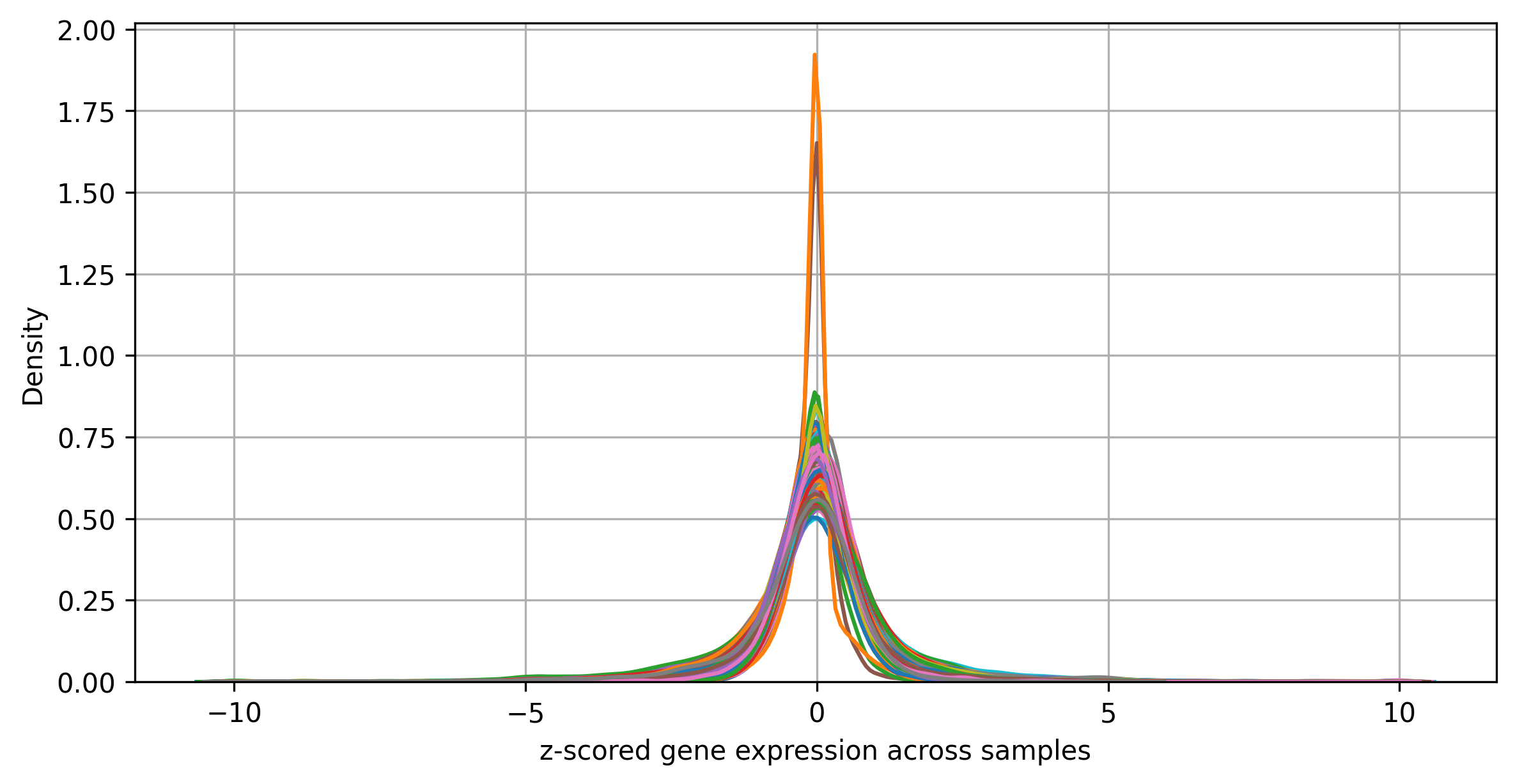

Supplement: S1 Fig — (TIFF) [file pcbi.1013188.s001.tiff]

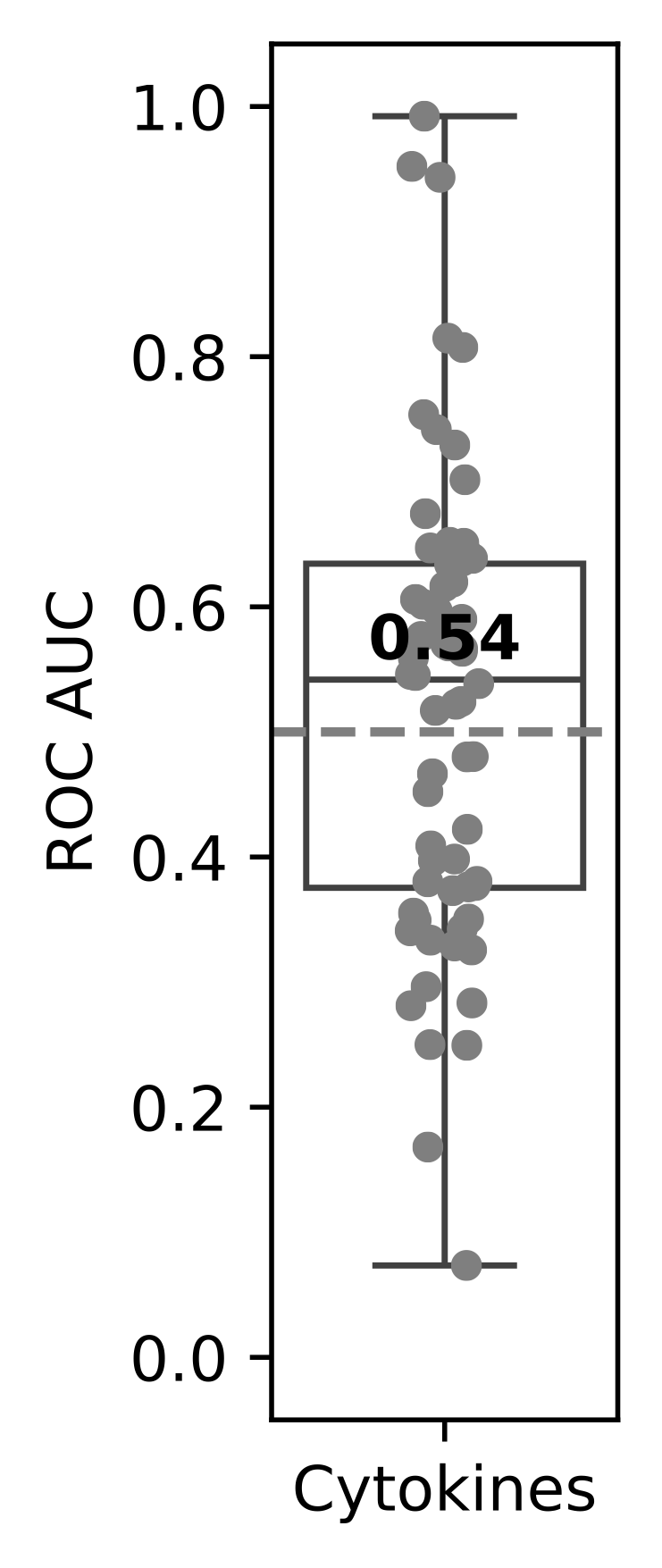

Supplement: S2 Fig — (TIFF) [file pcbi.1013188.s002.tiff]

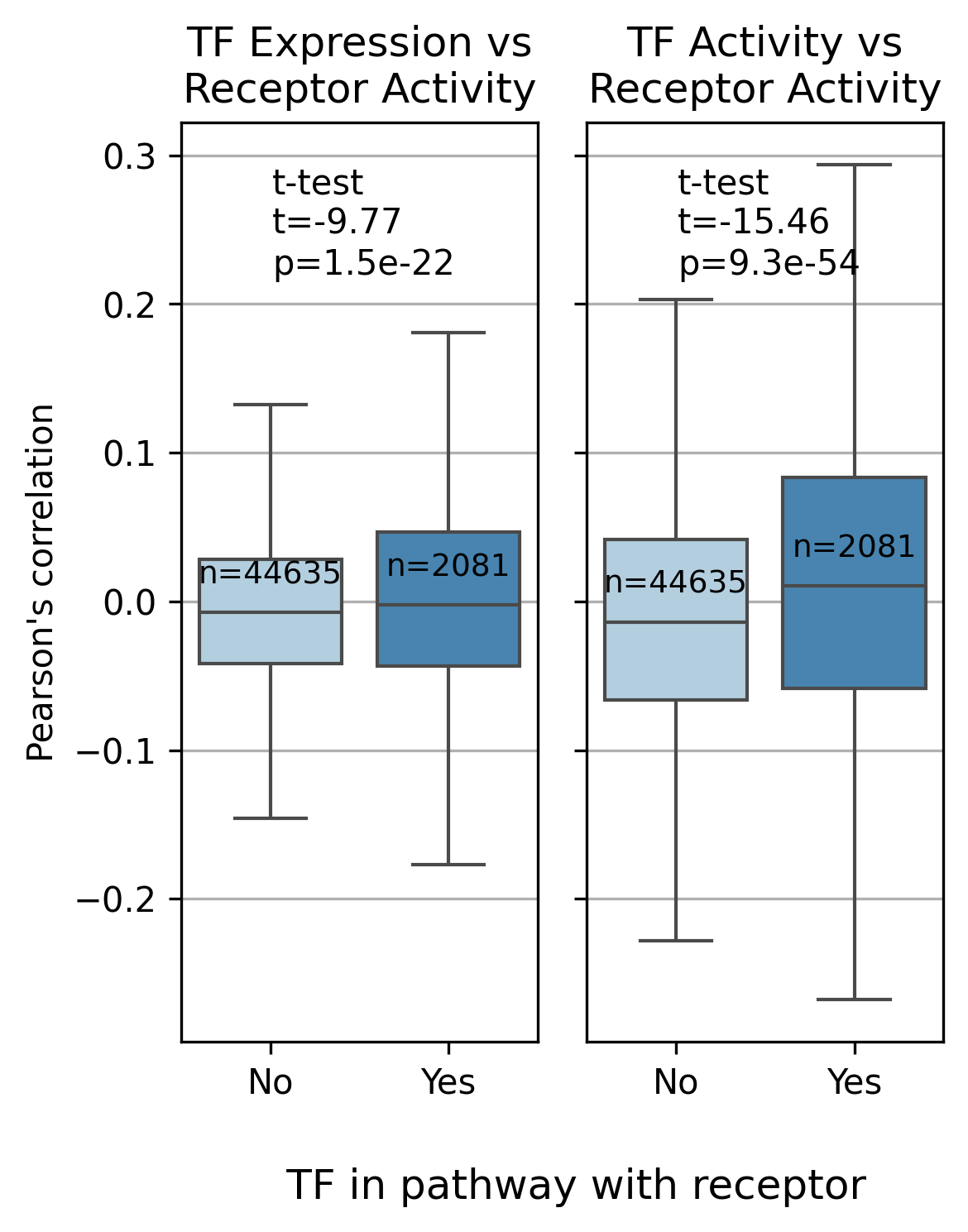

Supplement: S3 Fig — (TIFF) [file pcbi.1013188.s003.tiff]

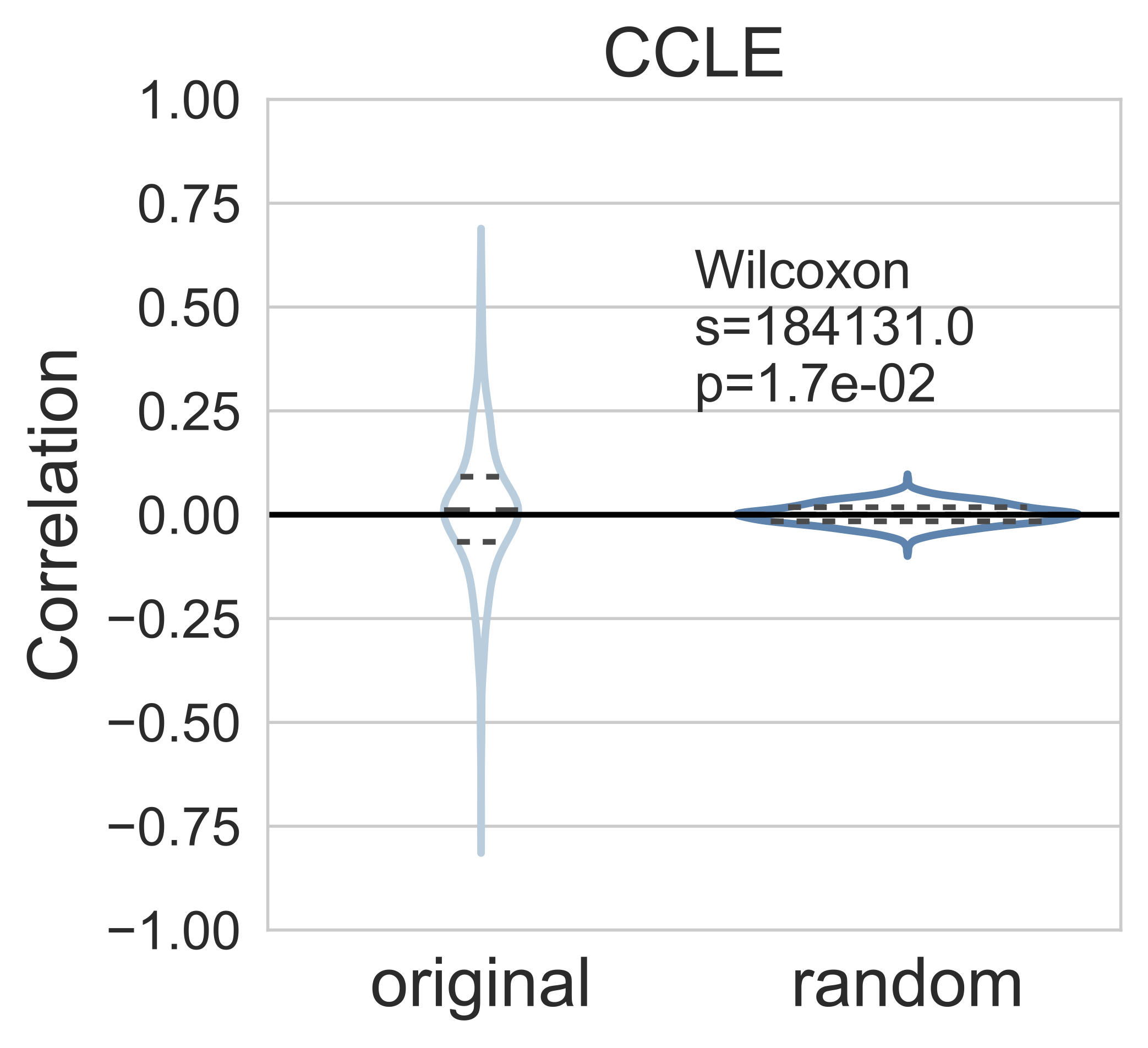

Supplement: S4 Fig — (TIFF) [file pcbi.1013188.s004.tiff]
