# Supplementary material for: RIDDEN: Data-driven inference of receptor activity from transcriptomic data
Source: PLoS Comput Biol. 2025 Jun 16;21(6):e1013188. doi: 10.1371/journal.pcbi.1013188 (PMC12193052; doi:10.1371/journal.pcbi.1013188)
Supplement: Attachment — Submitted filename: BarsiPLOSCB_2024_answers_to_reviewers.pdf [file pcbi.1013188.s011.pdf]

Revision

Reviewer #1

Comments to the Authors:

Thank you for your great effort on this work. Personally I really appreciate the novel application of RIDDEN on cancer therapy. However, I have a few comments on the methodology part.

1. First, why you chose Ordinary Least Squares (OLS) linear regression model to infer the association between receptors and other genes? OLS requires the error (residual) term follows normal distribution. Could you prove that? I know you could normalize the gene expression data to make them approximate to normal distribution, but the distribution of "y" in the regression model needs to be shown.

We thank the Reviewer for the insightful comments and the appreciation.

Regarding the model selection question, we applied Ordinary Least Squares (OLS) due to its computational efficiency, interpretability, and ability to handle large-scale gene expression data. Linear models have been successfully applied in similar studies, such as the PROGENy (Schubert et al., 2018) and CytoSig (Jiang et al., 2021), which is based on a collection of pathway-responsive genes and uses linear models to infer pathway activity. We applied the OLS models to estimate the relationship between gene expression and receptor perturbation, where the estimated coefficients indicate the association's strength and direction. An additional advantage is that we can identify generalist receptor-perturbation-induced changes, by averaging out the noise that is specific for the different cell types or experimental conditions.

We agree that to apply OLS, the data and the residuals should have approximate normal distribution. While raw gene expression data follows a negative binomial distribution, we used already preprocessed data from LINCS in our study. Level 5 data from LINCS is log-transformed and standardized, thus shows approximately normal distribution as shown in the figure below (**Response Figure 1**). Based on that we believe that the data distribution justifies the use of OLS.

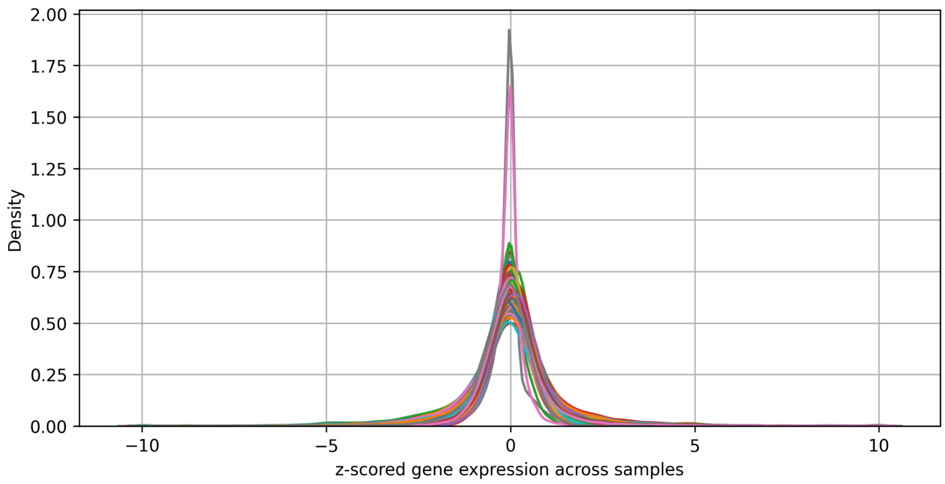

Response Figure 1. *Density plot showing the distribution of z-scored Level 5 LINCS data for gene expression across samples, with each line representing a single gene.*

2. Second, have you ever tried negative binomial regression model? Since negative binomial distribution is that best one to characterize gene expression profiles of transcriptomic data, can you try this one and check whether you get a better result? To construct a valid statistical model, it is essential to analyze the data distribution and select a model that accurately reflects it.

We thank the Reviewer for your thoughtful suggestion regarding using a negative binomial regression model. This is typically used for count data, such as RNA-seq read counts, due to its ability to the overdispersion in this RNA-Seq data. However, for our analysis, we are using LINCS z-scored Level 5 signatures (Subramanian et al., 2017) of perturbed samples. These signatures are continuous differential gene expression with standardized variance across genes. Considering this, we believe that other methods, such as Ordinary Least Squares (OLS), are more suitable for our analysis.

Data availability

We thank the Reviewer for the feedback on the availability of the signatures that serve as a basis for the RIDDEN model. The data is now available on Zenodo at the following DOI: 10.5281/zenodo.15127392. Additionally, we have updated the manuscript to include a 'Data Availability' section, which provides the DOI for this dataset.

Overall, this is a great and useful work. I recommend this work accepted after solving my comments above. Thanks.

We thank the Reviewer for the time and effort in understanding our study, and we appreciate the Reviewer's kind words, positive and constructive feedback, and thoughtful comments. We hope that our answers have addressed your questions and concerns.

Reviewer #2

The manuscript proposes a computational method, RIDDEN, to predict receptor activities from bulk and single-cell transcriptomics data. The assumption made in this manuscript is that the receptor activities are positively correlated with the target gene expression, which distinguishes this manuscript from the related cell-cell interaction prediction methods that focus on ligand and receptor gene expression. RIDDEN uses publicly available perturbation data to infer the target genes corresponding to each receptor, and use the inferred target gene expression to predict receptor activities of a new data. Despite the novelty of the idea, I have the following concerns.

We thank the Reviewer for the thoughtful review of our manuscript on RIDDEN. We appreciate the Reviewer's recognition of the novelty of our approach in predicting receptor activities from transcriptomics data.

1. **Even though the development of RIDDEN is motivated by that previous cell-cell interaction methods do not focus on the receptor side, RIDDEN is never compared against the existing cell-cell interaction inference methods based on both ligand expression and receptor expression. For example, the existing cell-cell interaction prediction methods can be applied to the Immune Dictionary dataset to predict whether each ligand-receptor pair is interacting, which can further predict whether a ligand/receptor is perturbed or not by checking whether it is interacting with any other receptors/ligands. This will allow a direct comparison with RIDDEN and CytoSig.**

In the Immune Dictionary (Cui et al., 2024), samples were stimulated with cytokines, and gene expression was measured after perturbation. This means that the ligands were added externally to the samples, so their expression is not directly altered and thus potentially not reflected in cytokine's gene expression signatures. Generally, cell-cell communication (CCC) methods consider that both the ligands and their receptors are present (expressed in the sender and receiver cells, respectively) and calculate the ligand-receptor interaction scores accordingly. Therefore, we do not expect high ROC AUC values when evaluating these CCC methods in this setup, particularly when assessing which receptors were activated in perturbed samples.

To test this hypothesis, we used LIANA (Dimitrov et al., 2022, 2024), a framework integrating existing CCC methods and knowledge, to compute CellPhoneDB (Efremova et al., 2020) ligand-receptor (LR) interaction scores. We evaluated cases where the receptor expression proportion is > 0.1 in the corresponding cell identities, but not filtered for the ligand expression since it was externally added in this study. We analyzed LR interactions between different single-cell clusters (B cells, macrophages, etc.) in response to the different perturbations. We calculated ROC AUC for every perturbation separately. We assigned a **1** to ligands that were perturbed in the sample and a **0** to those that were not. We then evaluated how well 1 - p-value, a measure of the specificity of the LR interaction, was predictive of the perturbed ligands in the sample.

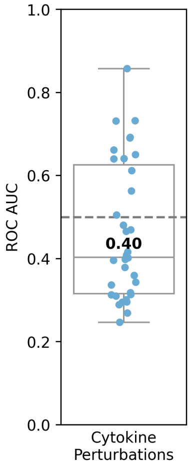

Response Figure 2. *ROC AUC evaluating CellPhoneDB's prediction of ligand perturbations in Immune Dictionary data*

Using the 44 overlapping cytokines (ligands) from CellPhoneDB and Immune Dictionary, we saw that performance (ROC AUC) of the LR interactions to differentiate between the cytokine stimulations is lower than 0.5 (**Response Figure 2**). This aligns with our expectations, as classical CCC methods are not necessarily designed to predict external stimuli. Therefore, we have decided not to include this comparison in the paper, as it may not provide a fair or meaningful evaluation. Instead, we added the following sentence to the discussion:

" Comparing RIDDEN with classical CCC methods on perturbation datasets would not yield meaningful insights, as these methods rely on endogenous ligand expression and are unable to capture the externally introduced ligands, making them unsuitable for accurately estimating interactions with receptors in perturbation experiments. "

2. In Fig 2C, "RIDDEN ALL overlap" and "CytoSig overlap" seem to have different number of points in the barplot. Why are they different on the "overlap" set?

We thank the Reviewer for the observation. The difference in the number of overlapping points arises from the prior knowledge of ligand-receptor interactions (OmniPath) that we apply, as well as the signaling components used in the two different methods. RIDDEN predicts receptor activities, while CytoSig predicts cytokine (ligand) signaling activities. There is no one-to-one correspondence between receptors and ligands; one receptor can bind to multiple ligands, and one ligand can bind to multiple receptors. In Fig 2C, we mapped receptors to multiple ligands and evaluated the performance of our predictions using the ROC AUC metric, which led to a higher number of points in the case of RIDDEN.

To improve the transparency of this, we extended the description of Fig. 2C.

"The first two boxes (RIDDEN ALL and CytoSig ALL overlap) represent the evaluation of models on the overlapping cytokines and their receptors of the three resources. **The cytokines of Immune Dictionary were mapped to receptors of RIDDEN, thus resulting in a different number of data points in this comparison.**"

3. AUROC value is sensitive to the class imbalance, and therefore the values of AUROC on different datasets may not be directly comparable. In the comparison between RIDDEN and CytoSig on each other's training datasets, I wonder what's the class imbalance of each training

datasets (percentages of label 0s and label 1s)? And if sub-sample each dataset to have the same class balance, what's the AUROC values are?

RIDDEN's receptor activities are evaluated on receptors, and CytoSig's cytokine signalling activities are evaluated on cytokines (negative and positive perturbations). We calculated the class imbalance ratios for receptors (RIDDEN) and cytokines (CytoSig) by dividing the number of positive samples (1) by the number of negative samples (0) for each receptor or cytokine (**Response Figure 3**).

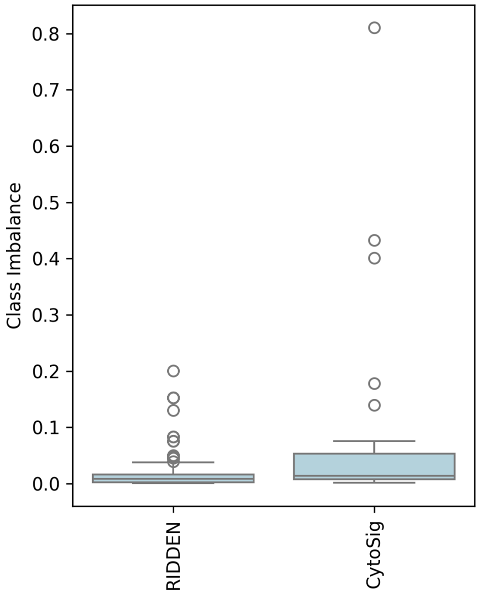

Response Figure 3. *Class imbalance ratios of RIDDEN and CytoSig, calculated as the ratio of label 1 samples (perturbation present) to label 0 samples (no perturbation) for each receptor or cytokine.*

We applied resampling with the following class ratios: 0.01, 0.05, 0.1, and 0.2, and we saw that changing the class ratios did not notably affect the results (**Response Table 1**).

Response Table 1. Model Performance on RIDDEN and CytoSig benchmark across different class ratios.

**class ratio**	**model**	**roc_auc**
0.01	CytoSig	0.582993
	RIDDEN	0.612279
0.05	CytoSig	0.584834
	RIDDEN	0.618610
0.10	CytoSig	0.593326
	RIDDEN	0.632792
0.20	CytoSig	0.589475
	RIDDEN	0.630382

4. When evaluating RIDDEN and CytoSig on each other's dataset, mapping cytokines to their receptors, and mapping receptors to the binding cytokines are used. I wonder how many cytokines/receptors have one-to-one mapping? If they don't have a one-to-one-mapping, how is the mapping handled exactly?

We thank the Reviewer for the insightful note regarding mapping cytokines to receptors and receptors to cytokines. We agree it is not clearly explained in the manuscript, so we have extended the mapping description for better understanding.

Briefly, in this analysis, we evaluate if the models (RIDDEN/CytoSig) can predict whether the cytokine receptors/cytokines were perturbed in the samples by assessing the predicted (receptor/cytokine signalling) activities using ROC AUC. We perform this analysis for every receptor to evaluate the RIDDEN and every cytokine to evaluate the CytoSig. We map the receptors to cytokines in the evaluation of RIDDEN predictions on the CytoSig's dataset (it can be one-to-one or one-to-more interactions), and we map the cytokines to receptors when we evaluate the CytoSig prediction on the RIDDEN's dataset.

Technically, in the case of RIDDEN evaluated on CytoSig's dataset using ROC analysis, we generated true label vectors for each receptor, where the true values were the cases where a cytokine has interaction with the given receptor based on the ligand-receptor interaction table of the OmniPath.

We followed the same approach for cytokines in the case of CytoSig evaluated on RIDDEN's dataset. However, this case was more complex because there are different perturbation types, such as compounds (which can be inhibition or activation and have single or multiple targets), shRNA, CRISPR (inhibitions) or stimulation, and overexpression (activation). Besides this, the perturbation of the sample can happen on the ligand or the receptor. Thus, we mapped all the perturbations (if it was a cytokine or cytokine receptor perturbation) to cytokines accordingly:

1) Inhibiting compounds - receptor perturbation: all the receptors mapped to their cytokines with a value of -1
2) Activating compounds - receptor perturbations: all the receptors mapped to their cytokines with 1
3) Stimulation - ligands perturbation (ligand stimulation/overexpression): the given ligand is labelled with a value of 1(no mapping required)
4) Inhibition - ligand perturbation (CRISPR, shRNA): the given ligand is labelled with a value of -1 (no mapping required)
5) Stimulation - receptor perturbation (overexpression) - All ligands of the perturbed receptor are labelled with a value of 1.
6) Inhibition - receptor perturbation (CRISPR/shRNA) - All ligands for the perturbed receptor are labelled with a value of -1.

We conducted separate analyses for cases with only activating perturbations, where all -1 were set to 0, and cases with only inhibiting perturbations, where all 1 were set to 0 and -1 were set to 1. Then, to reflect the more optimistic performance for CytoSig, we obtained the maximum values of the ROC AUC results for each cytokine from these two analyses. This section is described in Methods 4.6 and 4.4.

Methods 4.6. "…*We handled different perturbation directions separately (similarly as described in section 4.4.), calculating AUC values for stimulatory and 1-AUC values for inhibitory perturbations. The maximum ROC AUC values were selected for a cytokine with both types of perturbations*."

We extended the explanation of the mapping in Methods 4.6.

“... When evaluating RIDDEN on the CytoSig dataset, we mapped receptors to their interacting cytokines based on the ligand-receptor interaction table from OmniPath (Türei et al., 2016, 2021), allowing for both one-to-one and one-to-many interactions. When evaluating CytoSig on the RIDDEN dataset, we mapped cytokines to their corresponding receptors while accounting for the type of perturbation applied, which can be inhibitory or activatory.”

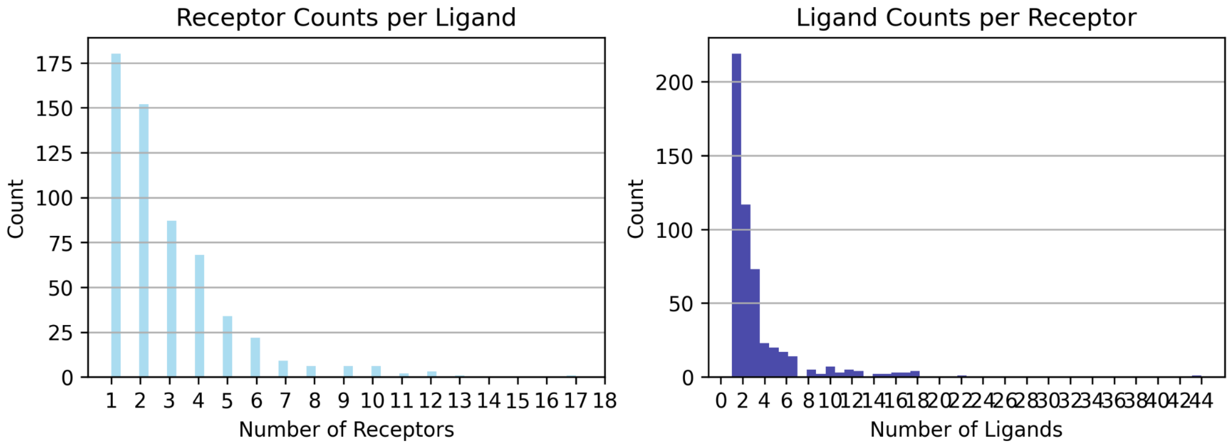

Response Figure 4. *Histogram of the number of receptors per ligand (left) and histogram of the number of ligands per receptor (right).*

5. What dataset is used for section 2.3 “Model evaluation and correlation with biology”?

In this section, we performed analyses, including receptor clustering performed on the RIDDEN model weight matrix, as well as receptor activity and expression correlations using TCGA and CCLE datasets. We assume the Reviewer is referring to the third analysis described in this section.

In this, we analyze the relationship between the receptors and transcription factor (TF) activity. We calculate the TF activities directly from the RIDDEN model’s weights. As model weights are corresponding to the strength (and direction) of receptor-regulated gene interactions, comparing this to TF gene regulatory interactions can reveal receptor-transcription factor signaling. To better highlight this in the manuscript now we write:

“Therefore, we estimated transcription factor activities of receptor model weights using decoupleR (Badia-I-Mompel et al., 2022) with TF regulons from DoRothEA (Garcia-Alonso et al., 2019). The RIDDEN model’s weights represent the receptor-induced gene expression signature.”

Technically, we applied decoupleR (Badia-I-Mompel et al., 2022) with TF regulons from DoRothEA (Holland et al., 2020) to estimate TF activities from the receptor model weights. These weights represent how gene expression changes in response to receptor perturbation. If genes in a TF’s regulon (i.e., genes regulated by the TF) have high weights, the corresponding TF activity is estimated to be high.

If receptor perturbation-associated genes overlap with TF-regulated genes, we assume that the TF is associated with the receptor and is likely downstream in the signaling pathway. This assumption allows us to infer whether TFs downstream of active receptors exhibit greater activity than those not involved in the same signaling pathways. To validate this, we compared the estimated activities of TFs that share KEGG signaling pathways with their associated receptors against those that do not.

6. In the analysis of TF expression and predicted receptor activities, I can think two other ways to strengthen the agreement between TF expression and RIDDEN prediction, besides what's shown in Fig 3B. (1) Whether TFs from the same pathway has a high weight in the RIDDEN receptor-gene weight matrix. (2) For each receptor and each TF in the same pathway, whether the cells/samples with low receptor activities and cells/samples with high receptor activity have differential gene expression of the TF. I suggest also include these analyses or providing a justification of why only consider the analysis in Fig 3B.

We appreciate the Reviewer's thoughtful suggestions for improving the validation of RIDDEN. While these ideas provide interesting perspectives, certain approaches may not be entirely feasible.

1) Whether TFs from the same pathway has a high weight in the RIDDEN receptor-gene weight matrix.

We assume there is no strong association between the TF gene expression and receptor activity, but rather with the TF-regulated genes, which the RIDDEN model captures.

We compared the available TF gene-receptor weights in the RIDDEN matrix, focusing on pairs within the same pathway versus those not part of it. We observed almost no difference in their weights (Response Figure 5). Additionally, the RIDDEN receptor-gene weight matrix is based on landmark genes, which limits the scope of this analysis.

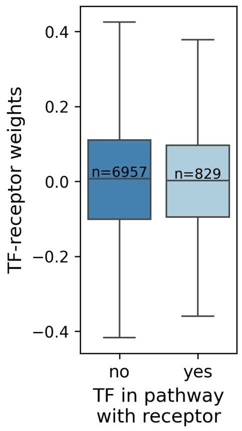

Response Figure 5. *Comparison of TF-receptor weights, showing TF-receptor pairs within the same pathway and those part of it.*

2) For each receptor and each TF in the same pathway, whether the cells/samples with low receptor activities and cells/samples with high receptor activity have differential gene expression of the TF. I suggest also include these analyses or providing a justification of why only consider the analysis in Fig 3B.

We appreciate the Reviewer's suggestion to further strengthen the validation of receptor-transcription factor relationships with transcription factor expression and receptor activity associations. To address this, we calculated receptor activity (by RIDDEN) and absolute TF activity (estimated using decoupleR (Badia-I-Mompel et al., 2022)) on CytoSig's dataset (Jiang et al., 2021) and we analyzed their correlation for pairs within the same KEGG pathway. The boxplot shows that receptor-TF pairs within the same pathway exhibit higher correlations compared to unrelated pairs. The result is similar to the previous approach, where we use the RIDDEN weights for comparison (**Response Figure 6**).

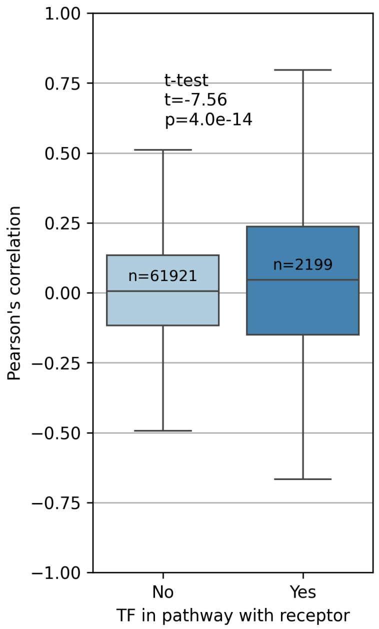

Response Figure 6. *Pearson Correlation between Receptor and TF activities when informed by intersect to KEGG or not.*

We included the following results in the manuscript in section 2.3.of the manuscript:

"We correlated receptor activity with transcription factor activity calculated on CytoSig's dataset, which showed that TF-receptor pairs within the same KEGG pathway exhibited higher activity than those from unrelated pathways (t-test p-value = $4x10^{-14}$)."

7. Regarding the methods.

(1). Ordinary least square is used to infer the receptor-gene weight matrix without regularization, which is the main matrix in RIDDEN and represents the effect on target genes of each receptor. However, OLS typically infers a dense coefficient vector, where almost all coefficients are nonzero, which indicate all receptors will have an effect on all genes. This doesn't seem correct biologically. In addition, dense coefficients may lead to overfitting to the training data and reduced generalization performance on new datasets. I suggest either trying a few regularization schemes or providing a justification of using the dense coefficient vector in OLS.
(2). The training data includes the perturbation of ligands and mapping the ligands to their corresponding receptors in the design matrix in OLS. However, ligand expression doesn't have a high correlation with the receptor expression nor receptor activities, which is the motivation of RIDDEN method and supported by result Fig 3C. I wonder whether including the ligand perturbation is necessary or not, if excluding these perturbations, what is the accuracy of RIDDEN?
(3). Does RIDDEN require a minimum number of perturbation experiments for each receptor?
(4). Section 4.1 uses the term "signature" for LINCS database, and this signature seems to have a different meaning than the gene expression signatures per receptor inferred by RIDDEN. What

does the "signature" mean here? And why "the receptor and ligand perturbation signatures" are filtered out instead of being retained?

1) OLS typically infers a dense coefficient vector, where almost all coefficients are nonzero…,

To address this question, we applied the Lasso regression method with an alpha value of 0.0001, where the independent variable (X) is a matrix of receptor perturbations (columns: receptors, index: genes and value: if the receptor was perturbed in the sample), and the dependent variable (y) is a vector of gene expression values across samples. In our dataset, we have 229 receptor features and 14,463 samples. Given this large number of samples relative to features, the risk of overfitting is lower, making OLS a reasonable approach despite its dense coefficient vector.

We utilized the same cross-validation technique described in Methods 4.5. For each receptor, we calculated the ROC AUC. The median ROC AUC using this approach is 0.71, which is consistent with the OLS method, where the ROC AUC is also 0.71 (**Response Figure 7**). Based on these results we believe that OLS has comparable performance to regularized methods on the RIDDEN dataset.

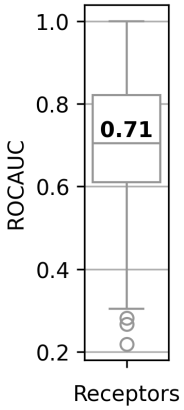

Response Figure 7. *ROC AUC values for cross-validation using Lasso regression.*

2) The training data includes the perturbation of ligands and mapping the ligands to their corresponding receptors in the design matrix in OLS. However, ligand expression doesn't have a high correlation with the receptor expression nor receptor activities, which is the motivation of RIDDEN method and supported by result Fig 3C. I wonder whether including the ligand perturbation is necessary or not, if excluding these perturbations, what is the accuracy of RIDDEN?

The Reviewer highlighted that RIDDEN is motivated by the observation that ligand expression does not always strongly correlate with receptor expression or activity, as shown in Figure 3C. However, in the LINCS database, there are experiments, where ligands are directly added as stimulation to the cells. In this case, we can reliably assume that these high concentrations of externally added ligands effectively stimulate their target receptors, so including them in RIDDEN model fitting is beneficial.

The RIDDEN dataset includes 1523 ligand stimulation signatures that interact with 173 out of 229 receptors in RIDDEN.

We saw that when these perturbation signatures were excluded from the data, creating a version of RIDDEN without them, the prediction performance on Immune Dictionary dropped from a median ROC AUC of 0.64 (Fig. 2C) to 0.53 (**Response Figure 8**). Thus, our results suggest that including ligand perturbations improves RIDDEN's accuracy on an independent dataset.

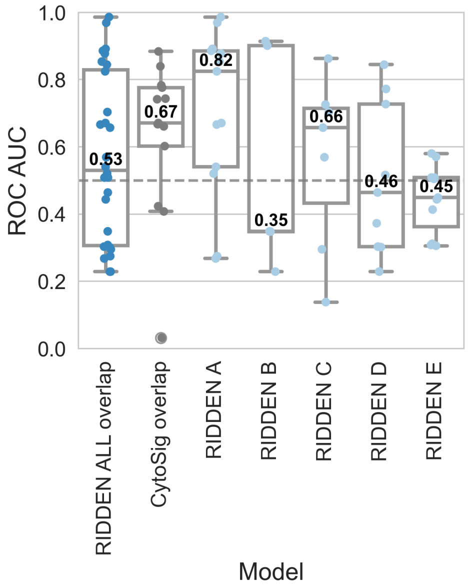

Response Figure 8. *ROC AUC values for different receptor activities of ImmuneDictionary evaluated using the RIDDEN model without ligand perturbation signatures*.

3) Does RIDDEN require a minimum number of perturbation experiments for each receptor?

We did not set a minimum threshold for the number of experiments for each receptor because the LINCS database provides a vast resource of perturbation data across several conditions and cell lines. RIDDEN utilizes the cell lines' consensus perturbation signatures (1 perturbation signature per cell line-perturbation pair), which ensures more experiments for each receptor. The final RIDDEN model is based on 14463 signatures covering 229 receptors. To enhance the reliability of the RIDDEN (receptor-gene weights), we implemented a framework where we validate receptor activity predictions using perturbations from other modalities.

"We first constructed 5 models for the 5 perturbation types (ligand, compound, CRISPR, etc.) separately, and we performed cross-validation to filter for receptors whose activity can be predicted at least in one other modality (See Methods). " Further details on this filtering process are provided in Methods section 4.4.

In this histogram, the number of experiments per receptor is shown (**Response Figure 9**). The authors of the PROGENy (Schubert et al., 2018), a similar method for inferring pathway activities, showed that the minimum required number of experiments varies between pathways, and the plateau effect was observed over 20 samples. In the RIDDEN, the minimum number of experiments is 7 for the CD27, 12 for the FSHR, and 210 out of 229 receptors have been involved in more than 20 experiments each.

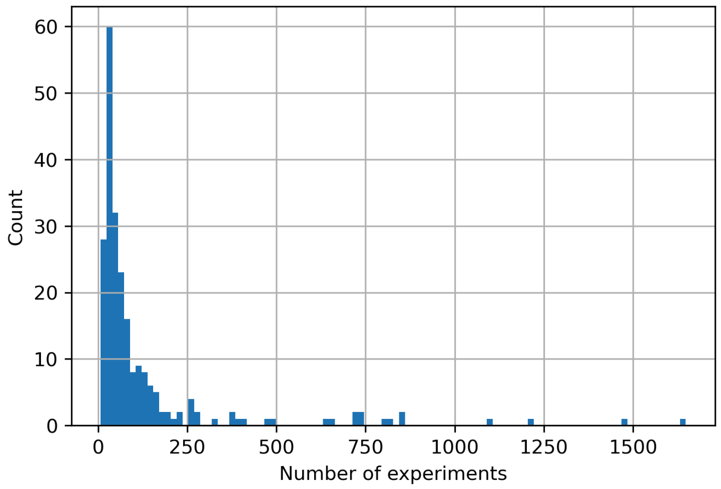

Response Figure 9. *ROC AUC values for different receptor activities of ImmuneDictionary evaluated using the RIDDEN model without ligand perturbation signatures*.

4) Section 4.1 uses the term "signature" for LINCS database, and this signature seems to have a different meaning than the gene expression signatures per receptor inferred by RIDDEN. What does the "signature" mean here? And why "the receptor and ligand perturbation signatures" are filtered out instead of being retained?

In Section 4.1, the term "signature" refers to the LINCS perturbation gene expression profiles, which capture transcriptional changes following a specific perturbation (e.g., ligand stimulation, receptor knockdown). We calculate consensus LINCS perturbation signatures by aggregating data across multiple experiments for each perturbation-cell line pair. This data serves as the foundation for the RIDDEN model, along with prior knowledge of ligand-receptor interactions. Using this data, we constructed the RIDDEN weight matrix, which encodes receptor-gene relationships. This matrix can be used to infer receptor activity from gene expression data.

And why "the receptor and ligand perturbation signatures" are filtered out instead of being retained?

Thank the Reviewer for this observation. The receptor and ligand perturbation signatures are indeed retained, and we have corrected this in the text.

*"From there, we **retained** all of the receptor and ligand perturbation signatures from the LINCS database from 5 modalities: … "*

Minor comments

Thank the Reviewer for the minor comments. We appreciate the Reviewer's attention to detail.

1. In Fig 1A, a transposition is needed of RIDDEN matrix to make the dimensions match in the matrix multiplication.

Fig 1A shows the linear model fit to generate receptor-gene weights from LINCS ligand and receptor consensus signatures (y) the prior knowledge (X). We fit one model on the expression of a gene across samples and the prior knowledge of whether the receptor was perturbed in the samples. Fig 1B shows the receptor activity inference, where we apply matrix multiplication, with dimensions of samples (n) x genes (m) and genes (m) x receptors (l), resulting in a samples (n) x receptors (l) matrix. We believe the current representation of matrixes describes our approach.

2. The results in Fig 1C, 2BC, and 3B need clarification.
1) Fig 1C indicates that the categorization of receptors into confidence levels can be performed using different types of perturbation experiments, and it's unclear how consistent the results are across them.

We agree that the description of Fig 1C in Results section 2.1. needs clarification.

First, we performed cross-validation to filter for receptors whose activity can be predicted in at least one other modality, which resulted in 229 receptors. This cross-validation and filtering are described in Methods 4.4. ("Improving the quality by signature filtering based on cross-validation").

We then performed a second cross-validation, now using all (14463) the signatures (across all modalities) to fit the RIDDEN for the 229 receptors. These receptors have several signatures across different modalities. In Fig. 1C, the last column ("Total") shows the total number of receptors (229) divided into different confidence levels, and the other columns show how these receptors are distributed across specific modalities and confidence levels. The process of assigning confidence levels to receptors is described in Methods 4.5. ("Assigning confidence levels to receptors").

When assigning confidence levels, we did not evaluate the receptors across different perturbation types. Instead, we focused on the overall predictive performance, measured by ROC AUC and the Mann-Whitney U test.

We have extended the main text to clarify this approach.

"..., we conducted an additional cross-validation and calculated the predictive performance for all 229 receptors, for which we used the complete set of their perturbation signatures from every modality (Fig 1C, Methods). This model (Fig 1C, labeled with Total) was then applied for subsequent analyses. The cross-validation resulted in a median ROC AUC score of 0.71. (Fig 1D)"

2) It's unclear which categorization (which x axis in Fig 1C) is used to generate Fig 2BC and Fig 3B.

The total number of receptors was predicted by RIDDEN. As we described in question 1, we fitted the model on all the signatures of the 229 selected receptors. We hope that the clarification provided in the Results section 2.1. enhances the understanding of our approach.

We thank the reviewer for taking the time and effort to read and review the manuscript in such detail and depth. We sincerely appreciate all your valuable comments and questions,

which helped improve the manuscript's quality. We hope that our response has thoroughly addressed your concerns and contributed to a better understanding of our work.

Reviewer #3:

Please see the attached review. In short, I recommend accepting this work after minor revisions.

In this work, Barsi et al. present a model, RIDDEN, a computational method to infer downstream receptor activation due to ligand-receptor binding that drives cell-cell communication. RIDDEN is effectively a weights matrix (genes x signal receptors) constructed from a curated range of perturbation data obtained from the LINCS 1000 database and prior knowledge obtained from the OmniPath database. Using this pre-trained matrix and additional post-processing, RIDDEN then predicts downstream receptor activity from transcriptomics data, either in the form or bulk or single-cell RNA-sequencing. The authors benchmark RIDDEN on ground truth receptor data against another method, CytoSig, demonstrating comparable accuracy and superior accuracy for the receptors inferred to be activated with the highest confidence level. The authors then apply RIDDEN to single-cell RNA-sequencing data of tumor samples, identifying that RIDDEN-inferred PD-1 receptor activity in pretreated patients correlated with better survival rates.

Overall, I find RIDDEN a pretty interesting and reasonable method that could be widely applicable. I think the use of LINCS L1000 perturbation data to construct the RIDDEN matrix in particular is a clever step. However, I would like to see some more comparisons of RIDDEN to other methods used for cell-cell communication to demonstrate its utility, and I think it would be interesting to apply RIDDEN to other types of data, e.g., Perturb-seq, CRISPR-based knockouts, spatial transcriptomics. Therefore, I recommend publication after minor revisions.

We would like to thank the Reviewer for the recommendation for acceptance of our manuscript. We greatly appreciate the Reviewer's insightful suggestions, which are helpful in improving the overall reliability of the RIDDEN method.

Major comments

1) The authors rightfully mention NicheNet as another method that, like CytoSig, tries to connect downstream gene expression to their potential upstream signal ligands, but do not compare RIDDEN to NicheNet. I understand NicheNet has somewhat different applications, but I think it would be worthwhile to see where the similarities and differences between NicheNet results and RIDDEN, especially as both rely on pretrained weights matrices to make predictions.

We used NicheNet to calculate ligand activity scores based on the transcriptomic response of cells to cytokine perturbations from the Immune Dictionary (Cui et al., 2024). We applied a sender-agnostic approach to evaluate all ligand-receptor (LR) interactions where the cognate receptors are expressed in the receiver cells. For each of the cytokine perturbations in the Immune Dictionary, we calculated the ligand (cytokine) activities for each single-cell type (acting as the receiver) This resulted in a dataset containing perturbation, cell type, ligand, and ligand activity information. Similarly to our approach in evaluating RIDDEN on ImmuneDictionary, we exclude perturbation-cell type pairs where the number of differentially expressed genes is ≤10.

To evaluate the performance of identifying the perturbed cytokine, we created true label vectors for each cytokine to indicate whether it was perturbed in the sample. ROC AUC was used to assess how well the ligand activity predicted the presence of the perturbed cytokine in the samples. Furthermore, we used

AUPR (Area Under the Precision-Recall Curve) as a metric for ligand activity, previously suggested as the most informative measure for defining ligand activity, particularly when evaluating the correlation between a ligand's target predictions and the observed transcriptional response (Browaeys et al., 2020). Finally, we evaluated 64 cytokine perturbations.

The median ROC AUC is 0.54 when evaluating the ligand activity per perturbation. (**Response Figure 10**) On the same dataset, RIDDEN achieved a 0.64 ROC AUC score (Figure 2C), although fewer cytokine (28) perturbations were evaluated due to a limited availability in transcriptomic data of these cytokines and their receptors in LINCS.

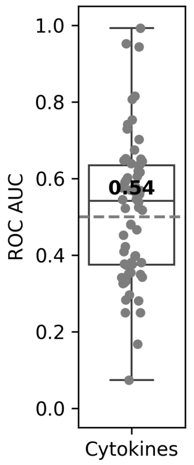

Response Figure 10. *ROC AUC values for evaluating ligand activity prediction on Immune Dictionary using NicheNet across 64 cytokine perturbations.*

We incorporated this analysis into the manuscript the Results Section 2.2:

"To compare the performance of RIDDEN to other ligand activity prediction methods, we also used NicheNet (NN) to predict ligand activity of Immune Dictionary cytokine perturbation data. The ligand activity predictions of the NN resulted in a median ROC AUC of 0.54, while evaluating 64 overlapping cytokines with Immune Dictionary. RIDDEN achieved a higher ROC AUC score on the three dataset overlap (Fig 2C), which included 28 cytokine receptors and their 58 interacting cytokines, 46 of which also overlapped with NN."

And Methods section *4.7: Benchmarking: NicheNet Model Ligand Activity Prediction Evaluation on the Immune Dictionary.*

And we included the figure as Supplementary Figure 1.

2) It would be worthwhile to compare RIDDEN to other cell-cell communication inference methods, such as CellChat, CellPhoneDB, LIANA, and others, to show that the receptor activities inferred by RIDDEN identify ligand-receptor interactions more accurately than the other methods, which only profile ligand and receptor gene expression.

For a meaningful comparison of cell-cell communication (CCC) methods, such as CellChat, CellPhoneDB, LIANA, with RIDDEN, or CytoSig, the ideal dataset should be a single-cell dataset where receptor activities or ligand-receptor interactions are measured that can be used as ground truth. While we are comparing CytoSig and RIDDEN on perturbation datasets like the CytoSig dataset and the Immune Dictionary, these datasets are not ideal for comparing RIDDEN to other CCC methods because they involve externally introduced ligands and are not present as expressed genes. In response to Reviewer #2's first question, we demonstrated that in the Immune Dictionary, a perturbation dataset, CCC methods cannot accurately estimate ligand-receptor interactions that align with our expectation, because the ligand is externally introduced (perturbation). When evaluating 64 ligands, the LR interactions were assessed using ROC AUC (where cells perturbed with the ligand were labeled as 1 and non-perturbed cells as 0), resulting in a ROC AUC score of 0.40 (**Response Figure 2**). Thus, to recap, we are not aware of any available dataset that provides both the necessary ground truth on receptor or ligand activation and avoids external perturbations, which are required for evaluating the performance of CCC methods accurately.

Minor comments

We appreciate the Reviewer's careful reading and helpful comments for improving our manuscript.

- **In the abstract and throughout the text, please change 'ligand bound receptors' to 'ligand-bound receptors'**

We corrected this in the abstract.

- **Throughout the text, please change 'ligand – receptor interactions' to 'ligand-receptor interactions'**

We corrected these in the manuscript text.

- **I noticed that, e.g., in the abstract, 'signaling' (US English) is used, while 'analyse' (UK English) is used. Either is fine, just as long as the text consistently US English only or UK English only.**

We switched the spelling to US English. We changed summarise to summarize and analyse to analyze.

We appreciate the Reviewer's time and effort dedicated to reviewing our manuscript in detail and providing insightful suggestions. We hope that our response addressed all the concerns raised. We thank the Reviewer for the positive feedback and recommendation for publication.

References

Badia-I-Mompel, P., Vélez Santiago, J., Braunger, J., Geiss, C., Dimitrov, D., Müller-Dott, S., Taus, P., Dugourd, A., Holland, C. H., Ramirez Flores, R. O., & Saez-Rodriguez, J. (2022). decoupleR: ensemble of computational methods to infer biological activities from omics data. *Bioinformatics Advances*, *2*(1), vbac016.

Browaeys, R., Saelens, W., & Saeys, Y. (2020). NicheNet: modeling intercellular communication by linking ligands to target genes. *Nature Methods*, *17*(2), 159–162.

Cui, A., Huang, T., Li, S., Ma, A., Pérez, J. L., Sander, C., Keskin, D. B., Wu, C. J., Fraenkel, E., & Hacohen, N. (2024). Dictionary of immune responses to cytokines at single-cell resolution. *Nature*, *625*(7994), 377–384.

Dimitrov, D., Schäfer, P. S. L., Farr, E., Rodriguez-Mier, P., Lobentanzer, S., Badia-I-Mompel, P., Dugourd, A., Tanevski, J., Ramirez Flores, R. O., & Saez-Rodriguez, J. (2024). LIANA+ provides an all-in-one framework for cell-cell communication inference. *Nature Cell Biology*, *26*(9), 1613–1622.

Dimitrov, D., Türei, D., Garrido-Rodriguez, M., Burmedi, P. L., Nagai, J. S., Boys, C., Ramirez Flores, R. O., Kim, H., Szalai, B., Costa, I. G., Valdeolivas, A., Dugourd, A., & Saez-Rodriguez, J. (2022). Comparison of methods and resources for cell-cell communication inference from single-cell RNA-Seq data. *Nature Communications*, *13*(1), 3224.

Efremova, M., Vento-Tormo, M., Teichmann, S. A., & Vento-Tormo, R. (2020). CellPhoneDB: inferring cell–cell communication from combined expression of multi-subunit ligand–receptor complexes. *Nature Protocols*, *15*(4), 1484–1506.

Garcia-Alonso, L., Holland, C. H., Ibrahim, M. M., Turei, D., & Saez-Rodriguez, J. (2019). Benchmark and integration of resources for the estimation of human transcription factor activities. *Genome Research*, *29*(8), 1363–1375.

Holland, C. H., Tanevski, J., Perales-Patón, J., Gleixner, J., Kumar, M. P., Mereu, E., Joughin, B. A., Stegle, O., Lauffenburger, D. A., Heyn, H., Szalai, B., & Saez-Rodriguez, J. (2020). Robustness and applicability of transcription factor and pathway analysis tools on single-cell RNA-seq data. *Genome Biology*, *21*(1), 36.

Jiang, P., Zhang, Y., Ru, B., Yang, Y., Vu, T., Paul, R., Mirza, A., Altan-Bonnet, G., Liu, L., Ruppin, E., Wakefield, L., & Wucherpfennig, K. W. (2021). Systematic investigation of cytokine signaling activity at the tissue and single-cell levels. *Nature Methods*, *18*(10), 1181–1191.

Schubert, M., Klinger, B., Klünemann, M., Sieber, A., Uhlitz, F., Sauer, S., Garnett, M. J., Blüthgen, N., & Saez-Rodriguez, J. (2018). Perturbation-response genes reveal signaling footprints in cancer gene expression. *Nature Communications*, *9*(1), 20.

Subramanian, A., Narayan, R., Corsello, S. M., Peck, D. D., Natoli, T. E., Lu, X., Gould, J., Davis, J. F., Tubelli, A. A., Asiedu, J. K., Lahr, D. L., Hirschman, J. E., Liu, Z., Donahue, M., Julian, B., Khan, M., Wadden, D., Smith, I. C., Lam, D., … Golub, T. R. (2017). A Next Generation Connectivity Map: L1000 Platform and the First 1,000,000 Profiles. *Cell*, *171*(6), 1437–1452.e17.

Türei, D., Korcsmáros, T., & Saez-Rodriguez, J. (2016). OmniPath: guidelines and gateway for literature-curated signaling pathway resources. *Nature Methods*, *13*(12), 966–967.

Türei, D., Valdeolivas, A., Gul, L., Palacio-Escat, N., Klein, M., Ivanova, O., Ölbei, M., Gábor, A., Theis, F., Módos, D., Korcsmáros, T., & Saez-Rodriguez, J. (2021). Integrated intra- and intercellular signaling knowledge for multicellular omics analysis. *Molecular Systems Biology*, *17*(3), e9923.
