# Supplementary material for: RIDDEN: Data-driven inference of receptor activity from transcriptomic data
Source: PLoS Comput Biol. 2025 Jun 16;21(6):e1013188. doi: 10.1371/journal.pcbi.1013188 (PMC12193052; doi:10.1371/journal.pcbi.1013188)
Supplement: Attachment — Submitted filename: BarsiPLOSCB_2024_answers_to_reviewers_2.pdf [file pcbi.1013188.s012.pdf]

Revision

Reviewer #1

1. The authors have provided a justification for the use of OLS regression, but does not add them in the manuscript.
From the figure in the response, the processed data from LINCS seem to follow normal distribution. It would be better if authors can show this model diagnosis plot as a supplementary figure to support the usage of OLS regression model. It would be perfect if hypothesis testing like Shapiro test, can be used to strictly show the data follows normal distribution.

We appreciate the Reviewer's suggestion to include model diagnostic plots to support the use of OLS regression. Therefore, we have added distribution plots of the processed LINCS data as a supplementary figure (Supplementary Fig 1).

2. The data used to build the model has been publicly available, but the link is not valid in the 'Data Availability' section.
I found that the data is now publicly available from the Github page of RIDDEN. However, the link provided by the author in 'Data Availability' section does not work.

Thank you for your comment. To enhance clarity and improve accessibility, we have updated the Data Availability section to explicitly include the link. We have also included this link in the README of the RIDDEN_analysis project on GitHub.

"The perturbation signatures of the receptors and the table of ligand-receptor interactions used to develop the RIDDEN model are available on Zenodo (DOI: https://doi.org/10.5281/zenodo.15127392). "

Minor comments

I suggest the authors consider revising the color scheme of the figures. Currently, most plots are based on blue tones, which may make it difficult to distinguish between elements or reduce visual impact. Incorporating a more diverse or contrasting color palette could improve clarity and readability.

We appreciate the Reviewer's input on the color scheme. After careful consideration, we have decided to retain the current palette. The blue tones were chosen mainly for design consistency. We believe the current scheme effectively conveys the information while providing a clean presentation.

Once these points are addressed, I would be happy to recommend acceptance.

We thank the Reviewer for the suggestions and careful revision. We hope that we have successfully addressed the points raised. We appreciate the Reviewer's recommendation and support for the manuscript's acceptance.

Reviewer #2

1. Regarding model evaluation using the relationship between receptor activity and TF activity, and RIDDEN model assumption between receptor activity and downstream gene expression change. It seems the assumption is that receptor activity is correlated with the downstream gene expression change, as well as TF "activity" change, but not TF gene expression change. Is that right? I suggest add a few sentences clearly state the assumption with justification.

We thank the Reviewer for this suggestion. Yes, the RIDDEN model assumes that receptor activity is associated with changes in the expression of downstream target genes, which may reflect changes in TF activity more than TF gene expression levels.

We expanded our previous analysis by examining correlations between receptor activity (as estimated by RIDDEN) and **absolute transcription factor (TF) activity** derived from the CytoSig dataset. In this updated analysis (**right** boxplot), we focused on receptor–TF pairs that belong to the same KEGG pathway and compared their correlation distributions to those of unrelated pairs. Additionally, we assessed the correlation between receptor activity and **TF expression levels** (**left** boxplot) for the same set of receptor–TF pairs.

Importantly, this updated analysis corrects two aspects of our prior approach: (1) we now compute correlations based on the **absolute** values of receptor and TF activity, rather than using signed values as in the previous version; and (2) we limit our analysis to receptor–TF pairs where receptor expression is available in the CytoSig dataset and TF activity was computed in the previous analysis using the decoupler tool. This resulted in a set of shared 204 TFs, compared to 280 in the earlier analysis.

The results demonstrate that receptor–TF pairs within the same pathway exhibit significantly higher correlations than those from different pathways, as demonstrated in earlier findings. Notably, this difference is more pronounced when comparing receptor activity with TF activity than when using TF gene expression, supporting the model's assumption (Response Figure 1).

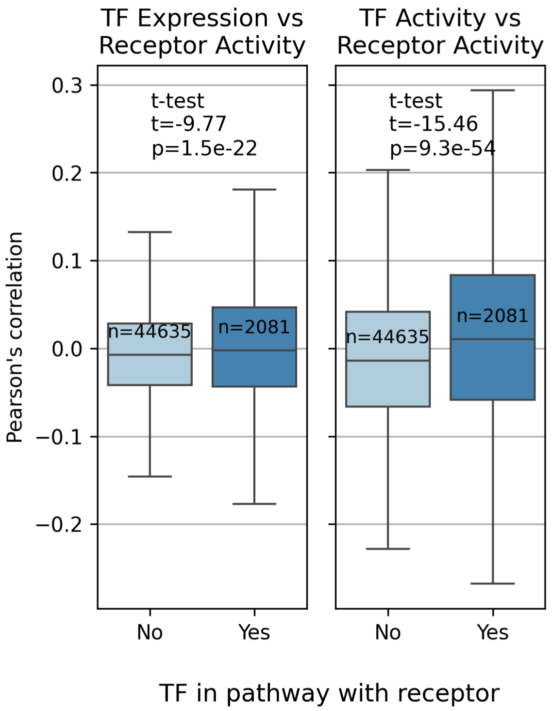

Response Figure 1. *Pearson Correlation between Receptor and TF activities or expression when informed by intersect to KEGG or not. Correlations are calculated using absolute values, and the analysis is limited to TFs with measured expression in CytoSig and available TF activity.*

We are also extending section 2.3. with the following:

"... RIDDEN infers receptor activity by capturing the gene expression changes induced by receptor perturbation. However, the gene expression does not necessarily reflect the activity of the proteins, therefore, the receptor-induced gene expression signature is expected to correlate more with the activity of downstream effectors, such as TFs, that directly regulate target genes in response to receptor signaling, rather than with the gene expression levels of those effectors themselves. Active receptors initiate signaling cascades that modulate the activity of downstream TFs…"

and

"... Additionally, we correlated receptor activity with both absolute TF activity and TF gene expression levels calculated from the CytoSig dataset. We found that the difference in correlation distribution between TF–receptor pairs within the same KEGG pathway versus unrelated pairs was substantially greater when using TF activity (t-test p-value = 9.3x10-54) compared to TF gene expression (t-test p-value = 1.5x10-22) (Supplementary Fig 3)."

Additionally, we extended the Methods section with a description of this analysis.

2. Regarding OLS with regularization, it's good to know that LASSO regularization doesn't change the performance of RIDDEN by a lot. But I think it's still important to

directly ensure RIDDEN weight matrix is biologically meaningful. I suggest looking into enriched pathways of up and down regulated genes of a few receptors based on RIDDEN weight matrix, and verify in previous literature whether the receptor is known to be related to the enriched pathways. As RIDDEN is a linear model, the high positive weight can be directly interpreted as up-regulation of the gene by receptor activation, vice versa for low negative weights. This evaluation also sheds light on the lack of information about the regulatory interactions for other signaling proteins than transcription factors, which was used as motivation in the intro.

We thank the Reviewer for this suggestion. In response, we performed enrichment analysis on genes with absolute weights in the RIDDEN weight matrix, focusing on a subset of receptors with distinct functions. Based on the top five significantly enriched biological process terms, the RIDDEN captures biologically meaningful downstream processes.

We have incorporated a description of this analysis into the manuscript in section 2.3.

Additionally, we extended the Methods section with a description of this enrichment analysis.

"We performed Gene Ontology biological process [1] enrichment analysis on gene weights of the RIDDEN receptors with distinct functions, to assess whether the model captures biologically meaningful patterns and to validate that genes with high absolute weights reflect relevant downstream processes of each receptor. As some representative examples, the top enriched terms aligned with known receptor functions, such as CXCR4 (C-X-C chemokine receptor type 4, a G protein-coupled chemokine receptor), which is associated with lymphocyte/leukocyte homeostasis [2]; IFNGR1 (interferon gamma receptor 1, a type II cytokine receptor) has a role in antigen processing and presentation [3]; IL6R (interleukin 6 receptor) os crucial for regulating immune responses as a cytokine receptor [4], and also plays a role in regulating autophagy [5]; TNFRSF1B (tumor necrosis factor receptor superfamily member 1B, a TNF receptor) is linked to T cell activation in immune response [6,7]; and TGFBR1 (transforming growth factor beta receptor 1, a TGF-beta receptor) is essential for all phases of wound healing [8,9], and is directly involved in hemostasis [10] (Supplementary Table 1). While enriched terms reflect receptor biology, complete overlap is not expected because methods like RIDDEN capture functional changes based on downstream effects rather than relying on predefined gene set enrichment."

	**CXCR4**	**IFNGR1**	**IL6R**	**TGFBR1**	**TNFRSF1B**
**1**	GOBP_LYMPHOCYTE_HOMEOSTASIS	GOBP_ANTIGEN_PROCESSING_AND_PRESENTATION_OF_EXOGENOUS_ANTIGEN	GOBP_POSITIVE_REGULATION_OF_RESPONSE_TO_CYTOKINE_STIMULUS	GOBP_NEGATIVE_REGULATION_OF_RESPONSE_TO_WOUNDING	GOBP_T_CELL_ACTIVATION_INVOLVED_IN_IMMUNE_RESPONSE
**2**	GOBP_LEUKOCYTE_HOMEOSTASIS	GOBP_NEGATIVE_REGULATION_OF_VIRAL_PROCESS	GOBP_SIGNALING_RECEPTOR_LIGAND_PRECURSOR_PROCESSING	GOBP_NEGATIVE_REGULATION_OF_WOUND_HEALING	GOBP_POSITIVE_REGULATION_OF_DNA_TEMPLATED_TRANSCRIPTION_INITIATION
**3**	GOBP_T_CELL_HOMEOSTASIS	GOBP_ANTIGEN_PROCESSING_AND_PRESENTATION_OF_EXOGENOUS_PEPTIDE_ANTIGEN	GOBP_SELECTIVE_AUTOPHAGY	GOBP_NEGATIVE_REGULATION_OF_COAGULATION	GOBP_LIPOPOLYSACCHARIDE_MEDIATED_SIGNALING_PATHWAY

**4**	GOBP_NEGATIVE_REGULATION_OF_B_CELL_APOPTOTIC_PROCESS	GOBP_ENDOTHELIAL_CELL_DEVELOPMENT	GOBP_REGULATION_OF_KERATINOCYTE_DIFFERENTIATION	GOBP_REGULATION_OF_COAGULATION	GOBP_CELLULAR_EXTRAVASATION
**5**	GOBP_B_CELL_APOPTOTIC_PROCESS	GOBP_ANTIGEN_PROCESSING_AND_PRESENTATION_OF_PEPTIDE_ANTIGEN	GOBP_EMBRYONIC_DIGIT_MORPHOGENESIS	GOBP_NEGATIVE_REGULATION_OF_NUCLEOTIDE_METABOLIC_PROCESS	GOBP_FEMALE_MEIOTIC_NUCLEAR_DIVISION

Response Table 1. *Table of Gene Ontology enrichment analysis for five receptors (CXCR4, IFNGR1, IL6R, TNFRSF1B, TGFBR1), based on the absolute gene weights derived from the RIDDEN model. The top five significantly (FDR < 0.05) enriched biological process terms are listed for each receptor.*

3. Regarding whether RIDDEN matrix should be transposed in Fig 1 or not. Fig 1B is correct, but in Fig 1A the matrix multiplication currently writes as perturbation profile (cells-by-genes) = design matrix (cells-by-receptors) * RIDDEN matrix (genes-by-receptors), which is incorrect in dimensions.

We appreciate the Reviewer for bringing this to our attention. The RIDDEN matrix dimensions in Fig. 1A have been corrected in the revised version.

We appreciate all of the Reviewer's efforts in improving our manuscript. We hope the corrections in the manuscript have addressed the Reviewer's concerns and hope that the Reviewer finds our manuscript suitable for publication.

References

1. Carbon S, Ireland A, Mungall CJ, Shu S, Marshall B, Lewis S, et al. AmiGO: online access to ontology and annotation data. Bioinformatics. 2009;25: 288–289.

2. Bianchi ME, Mezzapelle R. The chemokine receptor CXCR4 in cell proliferation and tissue regeneration. Front Immunol. 2020;11: 2109.

3. Zhou F. Molecular mechanisms of IFN-gamma to up-regulate MHC class I antigen processing and presentation. Int Rev Immunol. 2009;28: 239–260.

4. Wolf J, Rose-John S, Garbers C. Interleukin-6 and its receptors: a highly regulated and dynamic system. Cytokine. 2014;70: 11–20.

5. Hu F, Song D, Yan Y, Huang C, Shen C, Lan J, et al. IL-6 regulates autophagy and chemotherapy resistance by promoting BECN1 phosphorylation. Nat Commun. 2021;12: 3651.

6. Croft M. The role of TNF superfamily members in T-cell function and diseases. Nat Rev Immunol. 2009;9: 271–285.

7. Alam MS, Otsuka S, Wong N, Abbasi A, Gaida MM, Fan Y, et al. TNF plays a crucial role in inflammation by signaling via T cell TNFR2. Proc Natl Acad Sci U S A. 2021;118: e2109972118.

8. Penn JW, Grobbelaar AO, Rolfe KJ. The role of the TGF-β family in wound healing, burns and scarring: a review. Int J Burns Trauma. 2012;2: 18–28.

9. Ramirez H, Patel SB, Pastar I. The role of TGFβ signaling in wound epithelialization. Adv Wound

Care (New Rochelle). 2014;3: 482–491.

10. Jablonska E, Markart P, Zakrzewicz D, Preissner KT, Wygrecka M. Transforming growth factor-β1 induces expression of human coagulation factor XII via Smad3 and JNK signaling pathways in human lung fibroblasts. J Biol Chem. 2010;285: 11638–11651.

Reviewer #3

I am satisfied with the response to reviewer questions and comments and think the authors have done enough for the manuscript to be published.

We appreciate the Reviewer's feedback and are pleased that the revisions have addressed the concerns raised. We thank the Reviewer for supporting the acceptance of our manuscript.
