# Supplementary material for: RIDDEN: Data-driven inference of receptor activity from transcriptomic data
Source: PLoS Comput Biol. 2025 Jun 16;21(6):e1013188. doi: 10.1371/journal.pcbi.1013188 (PMC12193052; doi:10.1371/journal.pcbi.1013188)
Supplement: Attachment — Submitted filename: Barsi2025Review.docx [file pcbi.1013188.s010.docx]

**Review of Barsi et al., “RIDDEN: Data-driven inference of receptor activity from transcriptomic data”**

In this work, Barsi *et al.* present a model, RIDDEN, a computational method to infer downstream receptor activation due to ligand-receptor binding that drives cell-cell communication. RIDDEN is effectively a weights matrix (genes x signal receptors) constructed from a curated range of perturbation data obtained from the LINCS 1000 database and prior knowledge obtained from the OmniPath database. Using this pre-trained matrix and additional post-processing, RIDDEN then predicts downstream receptor activity from transcriptomics data, either in the form or bulk or single-cell RNA-sequencing. The authors benchmark RIDDEN on ground truth receptor data against another method, CytoSig, demonstrating comparable accuracy and superior accuracy for the receptors inferred to be activated with the highest confidence level. The authors then apply RIDDEN to single-cell RNA-sequencing data of tumor samples, identifying that RIDDEN-inferred PD-1 receptor activity in pretreated patients correlated with better survival rates.

Overall, I find RIDDEN a pretty interesting and reasonable method that could be widely applicable. I think the use of LINCS L1000 perturbation data to construct the RIDDEN matrix in particular is a clever step. However, I would like to see some more comparisons of RIDDEN to other methods used for cell-cell communication to demonstrate its utility, and I think it would be interesting to apply RIDDEN to other types of data, e.g., Perturb-seq, CRISPR-based knockouts, spatial transcriptomics. **Therefore, I recommend publication after minor revisions.**

Major comments:

- The authors rightfully mention NicheNet as another method that, like CytoSig, tries to connect downstream gene expression to their potential upstream signal ligands, but do not compare RIDDEN to NicheNet. I understand NicheNet has somewhat different applications, but I think it would be worthwhile to see where the similarities and differences between NicheNet results and RIDDEN, especially as both rely on pretrained weights matrices to make predictions.
- It would be worthwhile to compare RIDDEN to other cell-cell communication inference methods, such as CellChat, CellPhoneDB, LIANA, and others, to show that the receptor activities inferred by RIDDEN identify ligand-receptor interactions more accurately than the other methods, which only profile ligand and receptor gene expression.

Minor comments:

- In the abstract and throughout the text, please change ‘ligand bound receptors’ to ‘ligand-bound receptors’
- Throughout the text, please change ‘ligand – receptor interactions’ to ‘ligand-receptor interactions’
- I noticed that, e.g., in the abstract, ‘signaling’ (US English) is used, while ‘analyse’ (UK English) is used. Either is fine, just as long as the text consistently US English only or UK English only.
